# Oral Application of T4 Phage Induces Weak Antibody Production in the Gut and in the Blood

**DOI:** 10.3390/v7082845

**Published:** 2015-08-20

**Authors:** Joanna Majewska, Weronika Beta, Dorota Lecion, Katarzyna Hodyra-Stefaniak, Anna Kłopot, Zuzanna Kaźmierczak, Paulina Miernikiewicz, Agnieszka Piotrowicz, Jarosław Ciekot, Barbara Owczarek, Agnieszka Kopciuch, Karolina Wojtyna, Marek Harhala, Mateusz Mąkosa, Krystyna Dąbrowska

**Affiliations:** Institute of Immunology and Experimental Therapy, Polish Academy of Sciences, ul. R. Weigla 12, 53-114 Wrocław, Poland; E-Mails: joanna.majewska@iitd.pan.wroc.pl (J.M.); weronika.beta@iitd.pan.wroc.pl (W.B.); lecion@iitd.pan.wroc.pl (D.L.); katarzyna.hodyra@iitd.pan.wroc.pl (K.H.-S.); anna.klopot@iitd.pan.wroc.pl (A.K.); zuzanna.kazmierczak@iitd.pan.wroc.pl (Z.K.); pola@iitd.pan.wroc.pl (P.M.); agnieszka.piotrowicz@gmail.com (A.P.); jaroslaw.ciekot@iitd.pan.wroc.pl (J.C.); owczarek@iitd.pan.wroc.pl (B.O.); kopciuch@iitd.pan.wroc.pl (A.K.); karolina.wojtyna@gmail.com (K.W.); marek.harhala@gmail.com (M.H.); mateusz.makosa@iitd.pan.wroc.pl (M.M.)

**Keywords:** T4 phage, EBOV, Ebola virus, oral administration, vaccine, phage display, capsid proteins, antibodies, phage resistance

## Abstract

A specific humoral response to bacteriophages may follow phage application for medical purposes, and it may further determine the success or failure of the approach itself. We present a long-term study of antibody induction in mice by T4 phage applied *per os*: 100 days of phage treatment followed by 112 days without the phage, and subsequent second application of phage up to day 240. Serum and gut antibodies (IgM, IgG, secretory IgA) were analyzed in relation to microbiological status of the animals. T4 phage applied orally induced anti-phage antibodies when the exposure was long enough (IgG day 36, IgA day 79); the effect was related to high dosage. Termination of phage treatment resulted in a decrease of IgA again to insignificant levels. Second administration of phage induces secretory IgA sooner than that induced by the first administrations. Increased IgA level antagonized gut transit of active phage. Phage resistant *E. coli* dominated gut flora very late, on day 92. Thus, the immunological response emerges as a major factor determining phage survival in the gut. Phage proteins Hoc and gp12 were identified as highly immunogenic. A low response to exemplary foreign antigens (from Ebola virus) presented on Hoc was observed, which suggests that phage platforms can be used in oral vaccine design.

## 1. Introduction

Bacteriophages deliver a few important medical solutions. One of them is antibacterial therapy, which makes use of the natural ability of bacteriophages to kill bacteria. Currently, we are observing renewed interest in phage therapy as a promising alternative to antibiotics, mostly due to the problem of antibiotic resistance in bacteria. This inspires both recapitulation of previous experience and testing for an up-to-date methodology and approach [1,2,3,4,5,6,7]. Special regard is given to various aspects of phage interactions with organisms of treated individuals, since these interactions determine safety issues, phage pharmacokinetics, bioavailability and resulting outcomes of antibacterial treatment.

The other popular phage solution is the technological approach to phages as nanocarriers that are able to deliver biologically active elements. Nanocarriers may deliver various kinds of drugs, but they can also constitute a platform that allows for exposure of selected antigens. Such bacteriophage-based platforms are proposed as a new generation of safe (non-pathogenic) and effective vaccines. T4 phage capsid has been experimentally used to expose antigens of difficult pathogens, e.g., *Neisseria meningitidis* [8], anthrax [9,10] and HIV [11,12].

All medical applications of bacteriophages, including antibacterial therapy, vaccines and others, share a common feature: phages make direct contact with the mammalian organism and thus challenge the mammalian immunological system. One of the major consequences is a humoral response to a phage [13,14,15]. The humoral response, however, does not follow a simple schema of induction. It appears to depend on the route of phage administration and on individual features of a phage. It also depends on the dose and application schedule and possibly on other features, not yet specified [15,16,17,18]. As a consequence, it is not easy to draw a general conclusion about the effects that anti-phage antibodies have on the outcomes of therapeutic use of bacteriophages. Some authors found that the effect of the humoral response can be devastating [19], but others reported that anti-phage activity of serum does not exclude a favorable result of phage therapy in humans [17]. The first safety study of T4 phage application on humans revealed no antibody induction in phage-treated volunteers at all [2]. Difficulties with joint conclusions from different studies are related to the multi-factor nature of the immune system and its interactions with potential antigens.

Probably one of the most complex systems that can be considered in terms of therapeutic phage application is the gut, with its dynamic balance of symbiotic and sometimes pathogenic bacteria, natural and possibly therapeutic bacteriophages, as well as a variety of mammalian host-related factors. New technologies for sequencing and the metagenomics approach have revealed extreme microbiological diversity of the natural gut ecosystem, including bacteriophages [20,21]. This microbial balance, and especially its impact on human health, has been recently reviewed by Dalmasso *et al.* [22]. In spite of emerging interest in the topic, little is known about the humoral response to therapeutic bacteriophages if they are applied orally, even though it is generally expected that phages present in the gut may induce specific antibodies [16,23].

Here we present a long-term study (240 days) of specific antibody induction by T4 phage applied *per os* in a murine model: 100 days of phage treatment followed by 112 days without the phage, and then repeated treatment with the same phage up to day 240. The purpose of these studies was to provide immunological data useful for medical applications of bacteriophages, both those employing phages as antibacterials and those making use of phages as nanocarriers. In this work, the assessment comprised serum and gut antibodies (IgM, IgG, IgA) in relation to microbiological status of the animals: phage survival in gut and occurrence of phage-resistant bacteria. Immune response emerged as a major factor determining phage survival in the gut. The analysis concerned the individual impact of structural proteins on induction of the humoral response. Selected proteins were those exposed on the surface of phage head (gp23*, gp24*, Hoc, Soc) and the protein responsible for phage ability to infect bacteria (gp12). This allowed for the identification of highly immunogenic structural proteins and for further testing of the immune response to foreign antigen presented on the phage as a fusion with one of these proteins. Oligopeptides from Ebola virus served as exemplary foreign antigens that elicited immune response when displayed on T4 phage and administered *per os*.

## 2. Results

### 2.1. Induction of Anti-T4 Phage Antibodies in Mice Treated with the Phage per os

**Figure 1:**
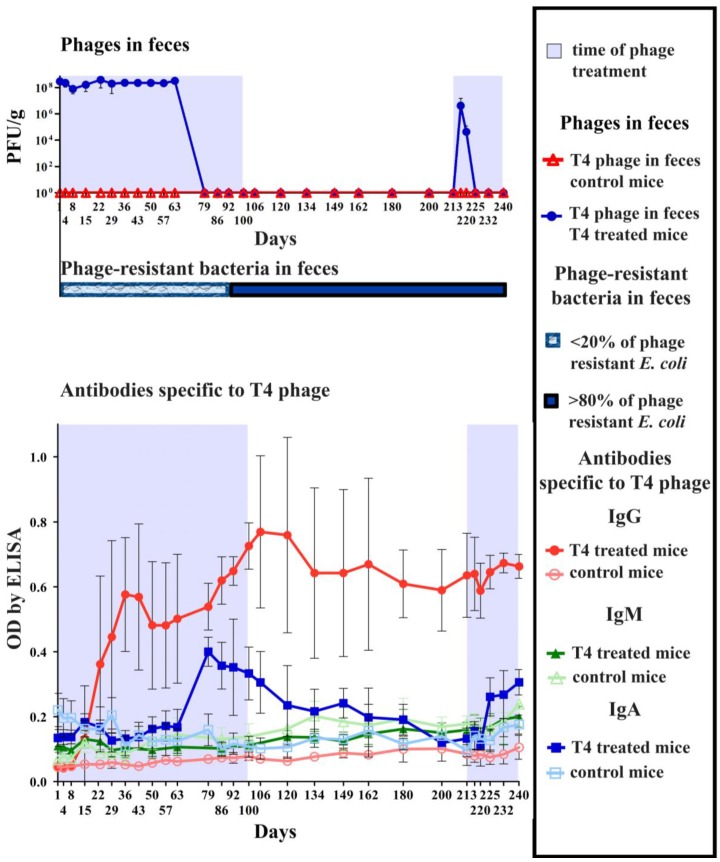
Bacteriophage T4 survival in the gut in correlation with increase of specific anti-phage antibodies and with emergence of phage-resistant *E. coli*. Mice (*N* = 7) were fed with T4 phage in drinking water to preclude micro-injuries that can be caused with a stomach probe and which result in false observations of phages penetrating the blood. The treatment was conducted for 100 days (days of phage treatment were marked in the figure with light blue). Then the phage was removed from the diet and the experiment was continued for the next 112 days. Then the phage was again administered to mice for 28 days (days of phage treatment were marked in the figure with light blue). Phage dose was 4 × 10^9^ pfu/mL with no pH-neutralizing additives (thus making approx. 2 × 10^10^ pfu/mouse daily as calculated from daily water uptake per animal). Control mice received no phage in the diet and they were separated from phage-treated mice. They were examined for presence of active T4 phage and no phage activity was detected during the whole experiment. IgG and IgM levels were tested in sera (blood was collected from the tail vein under anesthesia; thus the same mice were sampled for the whole experiment), and IgA levels were tested in feces. Additionally, feces were tested for the presence of active T4 phage and for T4 phage-resistant *E. coli*.

The long-term treatment and observation of mice took 240 days. During the first part, mice were given T4 phage in drinking water 4 × 10^9^ pfu/mL (approx. 2 × 10^10^ pfu per mouse daily) for 100 days, with no pH-neutralizing additives. Then the phage was removed from the diet and the experiment was continued for the next 112 days. Then the mice were again given T4 phage in drinking water with no pH-neutralizing additives 4 × 10^9^ pfu/mL (approx. 2 × 10^10^ pfu per mouse daily) up to day 240. Animals’ blood serum and feces were repeatedly tested for the level of specific anti-phage antibodies, during the whole 240-day period of the experiment. Serum samples were tested for IgG and IgM, and feces were tested for IgA. Animals were also monitored for the presence of active bacteriophages in feces and for possible occurrence of phage-resistant bacteria in feces; microbiological data were merged with immunological ones (presented in Figure 1).

As shown in Figure 1, the initial two weeks of continuous treatment with the phage had no significant effect on any class of investigated antibodies. From the third to fifth week (days 15–36), a constant increase of blood serum IgG antibodies was observed; IgG level increased markedly and statistically significantly (*p* < 0.001 in comparison to control mice on day 36). Contrary to expectations, no clear increase of IgM was noted before the IgG boost, and IgG level did not decrease after removal of the phage from the diet (after day 100). Secretory IgA increased considerably later, *i.e.*, approximately 7–8 weeks later than IgG (day 63–79). As a result, IgA reached its maximum after around two months of continuous treatment with the phage (*p* < 0.01 in comparison to control mice on day 79). Importantly, without continuous contact with the phage, secretion of specific IgA was gradually decreased, and on days 180–213 no significant difference was observed in comparison to control mice. The second exposure to the phage (starting from day 213) resulted in faster induction of secretory IgA: it was significantly increased in feces as soon as on day 225 (*p* < 0.05 in comparison to control mice) (Figure 1).

Phages were present in feces of all phage-fed mice as long as secretory IgA levels were low. Significant increase in IgA level on day 79 resulted in complete absence of active phages in feces. Interestingly, when secretory IgA decreased with time, repeated dose of the phage on day 213 resulted in recovery of active phages from feces (Figure 1). Active phages were detected until phage-specific IgA level increased again (day 225) (Figure 1).

Phage-resistant *E. coli* strains dominated the gut relatively late, *i.e.*, on day 92, shortly before the end of the phage treatment (Figure 1). From that date, they constituted 100% of bacterial colonies isolated from phage-treated mice, while in control mice only 20% of phage-resistant *E. coli* could be found in gut samples and 80% were sensitive to the phage. The prevalence of phage-resistant strains with small fluctuations was observed also during the whole time after removal of T4 from the diet and during the time of second treatment (up to day 240). No other substantial differences between phage-treated and control mice were detected in gut flora during the experiment.

**Figure 2:**
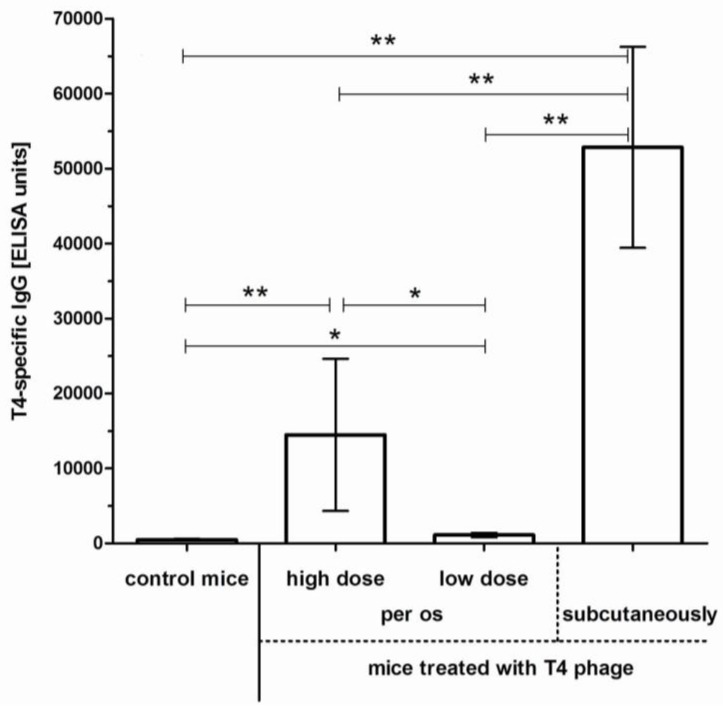
Intensity of anti-T4 phage IgG induction in mice treated with the phage *per os* and subcutaneously. Mice (*N* = 6 or 7) were fed with T4 phage in drinking water for 100 days, with no pH-neutralizing additives or they were injected with the phage subcutaneously. Two doses were used for the treatment *per os*: 4 × 10^9^ pfu/mL thus making approx. 2 × 10^10^ pfu/mouse daily, as calculated from daily water uptake per animal (in the Figure: high dose) or 4 × 10^8^ pfu/mL thus making approx. 2 × 10^9^ pfu/mouse daily, as calculated from daily water uptake per animal (in the Figure: low dose). Subcutaneous treatment was done with three subsequent injections: 5 × 10^9^ pfu/mouse on day 0, 5 × 10^9^ pfu/mouse on day 24, 2 × 10^9^ pfu/mouse on day 48, antibody level was tested on day 55. ELISA units were calculated for each sample according to Miura *et al.* [25,26]. Statistically significant differences between groups were marked with asterisks: * *p* < 0.05, ** *p* < 0.01.

In mice treated similarly with the same phage but in a lower concentration (4 × 10^8^ pfu/mL—one order of magnitude lower), the immunological response was very weak. A significant increase of secretory IgA was not detected (data not shown), which was correlated with the fact that the phage was constantly present in feces (10^5^ pfu/g); interestingly, phage-resistant *E. coli* did not dominate fecal samples even after 100 days. IgM induction was not detected in serum and the increase of serum IgG was meager and started later (day 30). Comparison of T4-specific IgG in murine sera on day 100 after phage treatment with 4 × 10^8^ pfu/mL to those after treatment with 4 × 10^9^ pfu/mL revealed 8.6 times higher immunization in the animals treated with the higher phage dose (Figure 2). These observations suggest that phage dose plays a major role in immunological outcomes of phage treatment. Serum IgG from mice treated with the phage *per os* for 100 days was also compared to that from mice injected with the phage s.c. (three doses, 5 × 10^9^ and 5 × 10^9^ and 2 × 10^9^ pfu/mouse). Injection, which typically results in a very effective delivery of phages to circulation [24], resulted in 5.5 higher immunization in comparison to the higher dose applied *per os* (Figure 2).

### 2.2. Individual Immunogenicity of Structural Proteins gp23*, gp24*, Hoc, Soc, and gp12 in Oral Application of T4 Phage

**Figure 3:**
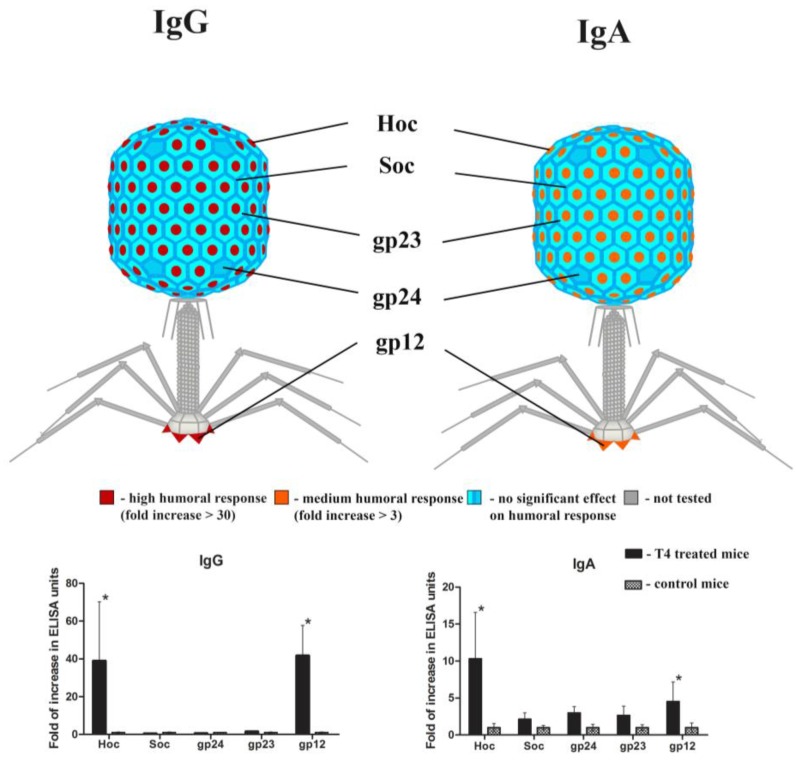
Individual contribution of T4 head proteins and gp12 to phage immunogenicity in mice treated with the phage *per os*. Mice (*N* = 7) were fed with T4 phage 4 × 10^9^ pfu/mL (thus making approx. 2 × 10^10^ pfu/mouse daily) in drinking water for 100 days, with no pH-neutralizing additives. Separated sera and feces from these mice were examined for IgG or IgA antibodies, respectively, specific to selected T4 structural proteins: gp23*, gp24*, Hoc, Soc, and gp12. ELISA units were calculated for each protein according to Miura *et al.* [25,26], fold of increase of ELISA units in comparison to control (non-immunized) mice was expressed by colors. Statistically significant differences were marked with asterisks.

Previous studies of T4 phage applied parenterally (i.p.) showed that phage structural proteins substantially differed in their individual immunogenicity [18]. Thus, specific response to the selected structural proteins (gp23*, gp24*, Hoc, Soc, gp12) was analyzed in samples from mice after 100 days of phage treatment *per os*. Antibodies specific to these proteins were analyzed by ELISA for the following classes: IgG in the blood and IgA in feces and results were expressed in ELISA units [25,26]. Proteins identified as significantly stimulating humoral response to the phage were graphically presented in Figure 3. A marked increase (in comparison to the control mice) was observed in anti-Hoc and anti-gp12 IgG (39- and 41-fold increase, respectively; *p* < 0.001). Similarly, anti-Hoc and anti-gp12 IgA were also significantly increased, but the increase was moderate (3.8- and 3.3-fold of increase, respectively; *p* < 0.05). Proteins Soc, gp23*, and gp24* were weakly immunogenic (Figure 3).

### 2.3. Immune Response to Foreign Antigens Presented as Fusions to Hoc Protein

T4 phage was previously demonstrated as a phage display platform applicable for presentation of foreign antigens, and those antigens were fused i.a. to the protein Hoc. Such modified phages were injected into the mice, and they elicited production of antibodies specific to presented foreign antigens [8,9,10,12]. Therefore, we further assessed potential ability of orally applied phages to serve as vaccine platforms. As exemplary foreign antigens, we used an oligopeptide from Ebola virus (here: antigen EB1). These oligopeptides were fused to Hoc and presented on T4 capsid by the competitive phage display [27]. Purified preparations of EB1-presenting phage were added to drinking water and mice were treated similarly as in the first part of the study. Mice were tested for specific anti-EB1 IgG and IgA; secretory IgA specific to EB1 was detected in the gut samples (Table 1).

**Table 1:** Induction of antibodies specific to the foreign antigen EB1 presented on T4 phage, after oral application of the phage. Mice were treated with the EB1 presenting phage in drinking water, control mice were not treated with phage or EB1 antigen, specific IgA and IgG levels were presented as mean ELISA units in groups (with SD); T4 phage specific antibodies served as a positive control of immunization. Statistically significant differences were marked with asterisks.

Class of Antibodies	Specificity of Antibodies	Type of Treatment	Immunization, ELISA Units	Statistical Significance (*p* < 0.05)
IgA	anti-EB1	T4_EB1 treatment	106,681 (±13,892)	*
		control	30,778 (±10,853)	
	anti-T4	T4_EB1 treatment	175,507 (±61,845)	*
		control	18,667 (±4628)	
IgG	anti-EB1	T4_EB1 treatment	282 (±47)	
		control	295 (±29)	
	anti-T4	T4_EB1 treatment	18,047 (±4655)	*
		control	557 (±24)	

## 3. Discussion

A specific humoral response to bacteriophages may follow phage application for medical purposes and it may further determine the success or failure of the approach itself. Therefore, we investigated specific antibodies induced by a model phage together with microbiological characteristics. Our studies of long-term phage treatment clearly show that phages applied *per os* are able to induce a humoral response. This response may emerge *in situ*, by secretion of specific IgA to the gut lumen, but also in the blood serum as specific IgG (Figure 1). Intensity of this response and the time necessary for its induction depend on the exposure to phage antigens, which is related to phage dose (Figure 2). Specific IgA secretion turned out to be a limiting factor for phage activity in the gut. Its significant increase correlated with the lack of phages detected in feces, while an increase of serum IgG did not significantly affect gastrointestinal transit of active phages. Phages were present in murine feces as long as secretory IgA levels were low. When IgA level increased on day 79, no active phages were detected in feces. Interestingly, secretory IgA decreased with time (on day 213 it was similar to its initial levels) and this decrease allowed for the successful passage of active phages through the gut for approximately one week. Active phages were detected until phage-specific IgA level increased again on day 225 (Figure 1).

Induction of serum IgG suggests that phage can be translocated from the gut lumen to the circulation. “Gut phage” ability to induce an immunological response in humans and in animals, or even their ability to penetrate to the blood and internal organs, has never been clearly defined. One may find both examples of phages that did [28,29,30,31] and those that did not [2,32] penetrate after gastric delivery. Some studies have shown that phage translocation was rather weak [33], but it was postulated to impact systemic immune reactions [16,34]. We detected a small titer of the phage (10^3^ pfu/mL) in murine blood after application of the higher phage dose (4 × 10^9^ pfu per mL of drinking water) (data not shown). The lower phage dose (4 × 10^9^ pfu/mL) did not allow for detectable translocation of the phage to the circulation (the detection limit was 200 pfu/mL of blood, as calculated from blood volume that was tested; this volume was limited due to ethical reasons). These observations show that even very low amounts of phage that reach the circulation can induce a long-lasting secondary response of the immune system, when the exposure is long enough.

For phage therapy purposes, it would be useful to assess general immunogenicity of the phage applied *per os* as “high” or “low”. This requires calculation of comparable phage doses between mice and humans. Pharmacokinetic scaling of bacteriophage doses from mice to humans is not easy, since typical calculation schema have been developed for much smaller agents of generally different characteristics (e.g., small proteins) [35]. Thus, to estimate adequate phage doses in humans we used the simplification of volumes as proportional to weight across species. With such a calculation, the higher dose of T4 phage used in this study for mice, 2 × 10^10^ pfu per mouse daily, equals 7 × 10^13^ pfu per human patient daily. Such high doses are, in the least, unusual in therapeutic approaches in humans; according to Sulakvelidze *et al.* [36], during the 20th century, phages were administered to humans orally, in tablet or liquid formulations containing 10^5^ to 10^11^ pfu/dose. Phage titers used for experimental therapy at the Institute of Immunology and Experimental Therapy (IIET), Phage Therapy Unit (Wrocław, Poland), in the years 2008–2010, ranged between 3 × 10^7^ and 6 × 10^10^ pfu per human patient daily [4]. Human volunteers in phage T4 safety tests reported by Bruttin and Brussow [2] received a total of 9 × 10^7^ pfu, and no specific antibodies were detected after that. In our studies, the lower, much less immunogenic dose applied to mice equals 7 × 10^12^ pfu per human patient daily, which is still much. Taking into account how persistent treatment was necessary to achieve a marked immunological response (two weeks of a higher dose to start the increase of IgG and more than two months to induce IgA), we conclude that T4 phage applied *per os* was weakly immunogenic.

Interestingly, these were immunological factors that turned out to limit phage viability in the gut, with no significant role of phage resistance in bacteria. Interestingly, among *E. coli* clones isolated from non-treated mice, as much as 80% were sensitive to T4 phage, which can be considered as a high fraction. Phage resistant bacterial strains dominated gut *E. coli* relatively late (day 92), and they did not determine the lack of viable phages in feces (Figure 1). This is in accordance with observations of Maura and Debarbieux [37], who reported that a phage present in the murine gut for 30 days did not give rise to domination of relevant phage-resistant bacteria. Possibly, the “arms race” between phages and bacteria is much more rapid in laboratory liquid monocultures than in the complex environment of the mammalian gut. Prolonged selection pressure necessary to select phage-resistant bacteria suggests that, at least in this model, phage propagation on commensal gut flora plays a marginal role. This is in line with the observations of Weiss *et al.* [38], who found it questionable whether T4 can effectively propagate on gut bacteria.

Structural elements of the phage capsid may differ in their individual immunogenicity [18]. Here we observed that humoral response to the phage was strongly stimulated by Hoc protein and gp12 (both IgG in the blood and IgA in the gut), while gp23*, gp24*, and Soc induced low response. High-level immunization against gp12 may strongly impact phage antibacterial activity, since gp12 plays a key role during phage infection of bacteria. A possible direction for future studies might be revealing factors promoting humoral response to gp12, which could be further applied for optimal design of phage use in humans and animals. The Hoc protein, in turn, was shown as an effective fusion protein in phage display of foreign antigens as presented by the group of V. Rao [8,9,10,12]. Here we proposed further study in the field; this study was comprised of a long duration of monitoring of the immune reaction and its dependency on dose, application schedule and route of administration. Recently, bacteriophages have also been employed for DNA vaccine technologies (BigDNA’s technology) that combined phages and DNA vaccines [39,40]. Thus, the high immunogenicity of Hoc may be of an advantage in developing vaccines. We found that foreign antigens displayed on the T4 phage as the Hoc-fusion can induce specific secretory IgA. We propose this result as an example of T4 phage use as an oral vaccine, and we hypothesize that further optimization of particular antigens and their arrangement on the capsid may deliver new solutions for vaccine development.

## 4. Materials and Methods

### 4.1. Bacteriophages

T4 phage was purchased from American Type Culture Collection (ATCC) (Rockville, MD, USA). The phage was cultured on an *Escherichia coli B* host obtained from the Collection of Microorganisms at the IIET, culture medium was LB-Broth high salt (Sigma-Aldrich, Poznań, Poland). The culture was conducted 8–10 h in 37 °C. Phage lysates were purified by filtration through polysulfone membrane filters 0.22 µm (Merck Millipore, Billerica, MA, USA) and added to drinking water for mice or used for further purification before they were applied as bottom antigens for ELISA. Purified phage preparations were obtained by two steps of chromatography: gel filtration on Sepharose 4B (Sigma-Aldrich, Poznań, Poland) followed by dialysis against PBS on 1000 kDa-pore membranes and LPS-affinity chromatography EndoTrap Blue according to the manufacturer’s instructions (Hyglos GmbH, Bernried, Germany). LPS removal was done by three successive incubations of the preparations with the slurry followed by centrifugations. The final samples were dialyzed against PBS, filtered with 0.22 µm PVDF filters (Merck Millipore, Billerica, MA, USA) and used for ELISA assay. Each purified phage preparation or phage lysate was tested for phage concentration by determination of phage titer after serial dilution with PBS (dilutions from 10^−1^ to 10^−9^). Fifty microliters of each dilution was spotted on a culture plate pre-covered with susceptible bacteria, three spots for each dilution. The plate was incubated for 8–10 h at 37 °C which was enough to obtain visible plaques. The plaques were counted, mean values of three spots were calculated and the phage concentration was calculated per milliliter with regard to the dilution and spot volume.

### 4.2. Bacteriophages Presenting Foreign Peptides

Bacteriophages presenting exemplary foreign antigen were prepared by competitive phage display as described previously [27]. Briefly, Ebola virus antigen from Zaire EBOV strain RWGFRSGVPPKVVNY [41,42,43] was used for phage display; this antigen was designed “EB1”. The relevant DNA sequence (CGTTGGGGCTTTCGTAGCGGCGTTCCGCCGAAAGTTGTTAATTAT) was cloned as N-terminal fusion with the gene *hoc* of T4 phage to expression vector pCDF-Duet-1 (Novagen, Merck Millipore, Darmstadt, Germany). The sequence coding for EB1 antigen was fused to the *hoc* gene using a mutagenizing primer in PCR. Expression *E. coli* B834 was transformed with the constructed plasmid, tested for effective production of EB1-Hoc fusion by SDS-PAGE and immunological detection, and used as a host for phage display cultures. Phage display cultures were conducted in LB-Broth high salt (Sigma-Aldrich, Poznań, Poland) in baffled flasks with shaking at 37 °C until OD600 was 0.08–0.1, induced with IPTG (final concentration: 0.05 mM) and incubated with shaking at 37 °C for 1 h. T4 phage was added to a final concentration of 10^5^ pfu/mL and incubated with shaking at 37 °C for 8 h. Lysate was clarified by centrifugation at 4000 g for 3 min, filtered with sterile 0.22 µm filters and purified (as described above). Final purified phage preparations were tested for the presence of EB1 antigen on T4 capsid by ELISA assay with a standard serum specific to synthetic EB1 oligopeptide (Lipopharm, Zblewo, Poland). Phage presenting EB1 on its capsid was designed by EB1-T4 phage.

### 4.3. Phage Proteins

Phage proteins were used as bottom antigens in ELISA immunoassay. They were produced as optimized by Miernikiewicz *et al.* [44,45]. Briefly, proteins were expressed in *E. coli* B834(DE3) F^−^ ompT hsdS_B_(r_B_^−^ m_B_^−^) gal dcm met (DE3) (Novagen, Merck Millipore, Darmstadt, Germany) grown in LB high salt (10 g/L of NaCl) (Sigma-Aldrich, Poznań, Poland). For expression of Soc and Hoc chaperone TF (from pTf16 vector, TaKaRa Bio Inc., Saint-Germain-en-Laye, France) and chaperones groES+groEL (from pGRO7 vector, TaKaRa Bio Inc., Saint-Germain-en-Laye, France) were used, respectively. Gp23* was co-expressed with gp31 chaperone of T4 phage. Gp12 was expressed from pCDF-Duet-1 with chaperone gp57 of T4 phage. Expression was induced with 0.2 mM IPTG (phage proteins) or 3 mM L-arabinose (TaKaRa chaperones) and conducted overnight at 25 °C. Harvested bacteria were lysed by freeze-thawing with lysozyme in phosphate buffer with PMSF (50 mM Na_2_HPO_4_, 300 mM NaCl, 1 mM PMSF, pH 7.5). The soluble fraction was incubated with glutathione sorbent slurry (Glutathione Sepharose 4B, GE Healthcare Life Sciences, Warsaw, Poland), washed with phosphate buffer, and proteins were released by proteolysis with AcTev protease (5 U/mL) (Invitrogen, Life Technologies Corporation, Waltham, MA, USA) at 10 °C; GST tags remained bound in the resin. In the case of gp12 Ni-NTA agarose was used without proteolysis. After intensive washing the protein was eluted with imidazole buffer. LPS removal from all protein preparations was done with EndoTrap Blue (Hyglos GmbH, Bernried, Germany). Gel filtration FPLC (fast protein liquid chromatography) on a Superdex 75 10/300 GL column (GE Healthcare Life Sciences, Warsaw, Poland) was applied and proteins were dialyzed against PBS and filtered through 0.22 µm PVDF filters (Merck Millipore, Darmstadt, Germany). Proteins were assessed by SDS-PAGE and concentrations were determined by the Lowry chromogenic method (Thermo Scientific, Rockford, IL, USA).

### 4.4. LPS Content Determination

The endotoxin level of the purified phage preparations was assessed using EndoLISA (ELISA-based Endotoxin Detection Assay, Hyglos, Bernried, Germany), according to the manufacturer’s instructions. Diluted samples or standard dilution with Binding Buffer were incubated overnight at room temperature with shaking. Subsequently, the plate was washed and Assay Reagent was added. The fluorescent signal was detected immediately by a fluorescence reader (Synergy H4 H4MLFPTAD BioTek Instruments, Winooski, VT, USA). This assay was used to determine LPS content in purified phage preparations serving as bottom antigen source in ELISA test. Phage preparation was used for ELISA when its LPS content was lower than 1 activity unit per mL in order to eliminate possible reaction of animal sera with residual LPS (false-positive reactivity of serum). If the LPS content was higher than 1 activity unit per mL, additional round of purification by LPS-affinity chromatography (described above) was conducted.

### 4.5. Immunization of Mice

C57Bl6/J male mice (6–16 weeks old) were used. The animals were bred in the Animal Breeding Center of the IIET, in specific pathogen free (SPF) conditions. Mice from phage-treated groups were separated from control mice. Food, water, litter, boxes and other accessories for all mice were sterilized.

Mice (N = 6 to 8) received phages in drinking water to prevent micro-injuries that can be caused by a stomach probe and which result in artificial introduction of phages into the blood. Phage concentration in water was (i) the lower dose 4 × 10^8^ pfu/mL (thus making approx. 2 × 10^9^ pfu/mouse daily, as calculated from typical daily water uptake) or (ii) the higher dose 4 × 10^9^ pfu/mL (thus making approx. 2 × 10^10^ pfu/mouse daily, as calculated from typical daily water uptake). Phage preparations were applied in drinking water without any additives for neutralization of stomach acidity, but water was mixed with PBS (1:1) in order to maintain proper ionic strength of the solution and to prevent phage aggregation and precipitation. Subcutaneous treatment was done with three subsequent injections: 5 × 10^9^ pfu/mouse on day 0, 5 × 10^9^ pfu/mouse on day 24, 2 × 10^9^ pfu/mouse on day 48, antibody level was tested on day 55. Feces and blood from the tail vein (under anesthesia, to heparinized tubes) were collected repeatedly during the experiment (*i.e.*, the same mice were sampled for the whole experiment). Feces were collected directly from mice (not as fecal pellets in the cages), diluted in PBS and tested for phage-specific IgA by ELISA (see below), as well as used for microbiological testing: samples were quantitatively cultured on selective microbiological plates with media selective for Gram-positive bacteria: Mueller Hinton II Blood Agar (30.0% beef infusion, 1.75% casein hydrolysate, 0.15% starch, 1.7% agar, 5.0% sheep blood, pH neutral), Gram-negative bacteria: MacConkey Agar (1.7% peptone, 0.3% proteose peptone, 1% lactose, 0.15% bile salts, 0.5% sodium chloride, 0.003% neutral red, 0.0001% crystal violet, 1.35% agar, pH 7.0–7.1) (Graso Biotech, Starogard Gdański, Poland), and for bacteriophages: Mueller Hinton II Agar (30.0% beef infusion, 1.75% casein hydrolysate, 0.15% starch, 1.7% agar, pH neutral with *E. coli* B host layer) (Graso Biotech, Poland). Identification of *E. coli* isolated from feces was confirmed by automated mass spectrometry microbial identification system (VITEK^®^MS, Biomerieux, Durham, NC, USA). *E. coli* isolated from feces were tested for their sensitivity to T4 phage infection (60 colonies) by culturing of bacterial monolayers on culture plates with phage preparations spotted on the top; plaque formation was assessed. Serum for IgM and IgG testing was separated from the blood by double centrifugation (2250 *g* and 10,000 *g*), each time cell and platelets pellet was discarded and supernatant fraction was saved. After the second centrifugation it was used for ELISA assay. All experiments were repeated 2–4 times. One exemplary experiment of each type was presented. Each timepoint represents multiple mice, timepoints were the same for antibody testing and for microbiological testing.

### 4.6. Ethics Statements

All animal experiments were performed according to EU Directive 2010/63/EU for animal experimentations and were approved by the 1st Local Committee for Experiments with the Use of Laboratory Animals, Wroclaw, Poland (No. 64/2009 and 76/2011). The authors followed the ARRIVE (Animal Research: Reporting of *in vivo* Experiments) guidelines [46].

### 4.7. Specific Antibody Level Measurement by ELISA

A MaxiSorp flat-bottom 96-well plate (Nunc, Thermo Scientific, Poznań, Poland) was covered with purified phage preparations obtained by chromatography as described above (100 µL per well, 5 × 10^9^–10^10^ pfu/mL) or proteins (100 µL per well, 10 µg/mL) or oligopeptide EB1 (100 µL per well, 80 µg/mL) sterilely, at 4 °C, overnight. Plates were washed 5 times with PBS and blocked with 1% albumin for 1 h (100 µL per well) at room temperature. Albumin was removed and the plate was washed 5 times with PBS with 0.05% Tween 20 (Serva, Heidelberg, Germany). Serially diluted serum was applied to the wells in 100 µL per well. In the case of secretory IgA detection in feces, feces were homogenized in PBS in proportion 1/10, *i.e.*, each 0.1 g of feces was supplemented with PBS up to 1 mL. and then serially diluted and applied to the wells in 100 µL per well. Samples from each mice were processed separately (sera/feces were not pooled in the groups). Each sample was processed in duplicate. The plate was incubated at 37 °C for 2 h. Plates were washed 5 times with PBS with 0.05% Tween 20 (Serva, Heidelberg, Germany). Diluted detection antibody was added in the amount of 100 µL per well: peroxidase-conjugated AffiniPure goat anti-mouse IgM (Jackson ImmunoResearch Laboratories, West Grove, PA, USA) or peroxidase-conjugated AffiniPure goat anti-mouse IgG (Jackson ImmunoResearch Laboratories) or peroxidase-conjugated AffiniPure goat anti-mouse IgA (Jackson ImmunoResearch Laboratories). Detection antibody was incubated in the wells for 1 h at RT in the dark, removed, and the plate was washed 5 times with PBS with 0.05% Tween 20 (Serva, Heidelberg, Germany). TMB X-Treme substrate reagent for peroxidase was used (50 µL) according to the manufacturer’s instructions (ImmunO_4_, Westminster, MD, USA). Twenty-five microliters of 2N H_2_SO_4_ was added to each well without substrate removal, then absorbance was measured at 450 nm (main reading) and 550 nm (background). The background values were subtracted from the main readings and the average value of each duplicate was calculated. In the case of relative increases of antibody levels in time (Figure 1), OD values were presented. To compare immunogenicity in selected conditions, to compare individual immunogenicity of phage proteins and to assess immunization by EB1 antigen (Figure 2 and Figure 3, Table 1), highly responsive sera from animals were used to establish a reference standard serum according to Miura *et al.* [24,25]. Briefly, to calculate ELISA units (EU), 10 points of standard serum dilutions were determined on each plate, and the dilution giving an optical density at 450 nm (OD450) of 1 was assigned as 1000 EU. A standard curve was calculated by the plate reader software (Gen5 Data Analysis Software) and fitted to a function that converted OD values to EU. Each EU value was normalized, and the average value of each duplicate (per sample) was calculated. Immunogenicity in selected conditions was assessed by fold of increase in EU (comparing immunized animals to control animals). All experiments were repeated 2–4 times; they were not summarized; exemplary experiments with their individual N values and statistical significance were presented. Statistical analysis was done by one-way analysis of variance (ANOVA, Tukey) or the Kruskal-Wallis test or the Mann-Whitney test with the Statistica 8.0 software package [47].

## 5. Conclusions

Phages present in the gut can induce anti-phage antibodies in blood when the exposure is long enough. The effect is also dose dependent. T4 phage (used in this study) appeared to be low immunogenic, since only very high doses of the phage applied for a long time elicited a significant increase in specific antibody levels. However, secretion of specific IgA, even if not readily induced, had a devastating effect on phage viability. Increase of IgA seemed to play a much more important role than selection for phage-resistant *E. coli* in the gut flora; phage-resistant strains occurred relatively late. Termination of phage treatment results in a gradual decrease of secretory IgA, even to insignificant levels that again allow for transition of active phage particles through the gut. Second administration of phage also induces secretory IgA, it increases sooner than that induced by the first administration. Specific antibodies in the blood and in the gut are induced by proteins Hoc and gp12, while gp23*, gp24*, and Soc are weakly immunogenic. Foreign antigens presented on the phage can also induce antigen-specific antibodies, thus we propose T4 phage as possible platform for oral vaccines development.

## Supplementary Files

Supplementary File 1
