# Supplementary material for: Oral Application of T4 Phage Induces Weak Antibody Production in the Gut and in the Blood
Source: Viruses. 2015 Aug 20;7(8):4783–99. doi: 10.3390/v7082845 (PMC4576206; doi:10.3390/v7082845)
Supplement: Supplementary File 1 [file viruses-07-02845-s001.pdf]

Supplementary Materials

Ebola virus antigen from Zaire EBOV strain RWGFRSGVPPKVVNY (EB1) was used as an exemplary foreign antigen and it was presented on T4 capsid by competitive phage display [27] (in the Figure: EB1-T4). The phage was purified and tested for the presence of EB1 antigens by ELISA, in comparison to a control phage modified with 6-histidine sequences instead of EB1. Additionally, solving buffer from purified phage preparation was separated on 100-kDa membranes and tested for the presence of EB1 antigens by ELISA (in the Figure: background); this was done to exclude potential remains of EB1 unincorporated to the phage capsid.

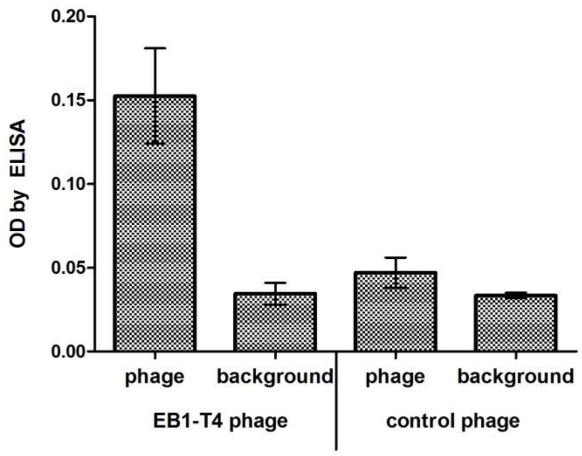

Figure S1. Purified preparation of T4 phage modified with oligopeptides EB1 derived from Ebola virus: immunological identification of EB1 oligopeptides on the phage capsid and in the solvent.
